# Anti-Inflammatory and Antioxidant Effects of HIIT in Individuals with Long COVID: Insights into the Potential Role of *Triphala*

**DOI:** 10.3390/ijms26178623

**Published:** 2025-09-04

**Authors:** Tadsawiya Padkao, Suwipa Intakhiao, Nattaphol Prakobkaew, Surachat Buddhisa, Yothin Teethaisong, Orachorn Boonla, Piyapong Prasertsri

**Affiliations:** Faculty of Allied Health Sciences, Burapha University, Chonburi 20131, Thailand; tadsawiya@go.buu.ac.th (T.P.); suwipa.in@go.buu.ac.th (S.I.); nattaphol@go.buu.ac.th (N.P.); surachat.bu@go.buu.ac.th (S.B.); yothin.te@go.buu.ac.th (Y.T.); orachorn@go.buu.ac.th (O.B.)

**Keywords:** exercise therapy, phytotherapy, inflammation, oxidative stress, long COVID

## Abstract

Long COVID is characterized by persistent symptoms associated with chronic inflammation and oxidative stress. While high-intensity interval training (HIIT) and supplementation with antioxidants such as *Triphala* have demonstrated individual therapeutic benefits, their combined effects remain unclear. This study aimed primarily to evaluate the effects of an 8-week HIIT program on markers of inflammation, oxidative stress, and exercise-related symptoms in individuals with long COVID, and secondarily to explore whether *Triphala* supplementation provided additional benefits. A total of 104 participants (aged 18–59 years) were randomized into three groups—control (placebo), HIIT (cycling for 28 min/day, 3 days/week), and combined (HIIT + *Triphala*, 1000 mg/day)—for 8 weeks. The biomarkers assessed included interferon-gamma (IFN-γ), tumor necrosis factor-alpha (TNF-α), malondialdehyde (MDA), protein carbonyls, and superoxide dismutase (SOD) activity. Following the intervention, significant reductions in IFN-γ, TNF-α, MDA, protein carbonyls, and rating of perceived exertion were observed in both the HIIT and combined groups (*p* < 0.05), with no significant differences between the two. SOD activity significantly increased in all groups, including the control group (*p* < 0.05), with no between-group differences. An 8-week HIIT program appears to be effective in reducing inflammation, oxidative stress, and dyspnea in individuals with long COVID. *Triphala* supplementation did not provide any additional statistically significant benefit but was safe and well tolerated.

## 1. Introduction

Coronavirus disease 2019 (COVID-19) remains a significant global public health concern, with the number of confirmed cases continuing to rise worldwide according to the World Health Organization (2025) [1]. During the pandemic, a growing number of individuals reported persistent or relapsing symptoms following acute infection with severe acute respiratory syndrome coronavirus 2 (SARS-CoV-2). These prolonged symptoms are now collectively termed “post-COVID-19 condition” or “long COVID”. Long COVID is a chronic, infection-associated condition that develops after SARS-CoV-2 infection and persists for at least three months; it may follow a continuous, relapsing–remitting, or progressive course, potentially affecting one or more organ systems [2,3]. Current epidemiological estimates suggest that approximately 10% of individuals who recover from acute infection go on to develop long COVID [4].

The precise pathophysiological mechanisms underlying long COVID remain incompletely understood. Several hypotheses have been proposed, including immune dysregulation, the persistence of viral components that sustain chronic inflammation, endothelial dysfunction or activation, microvascular thrombosis, mitochondrial impairment, metabolic disturbances, and unresolved tissue injury [4,5]. Hyperactivation of innate immune cells—such as T cells, natural killer cells, and mast cells—can trigger the release of chemokines and pro-inflammatory signals, ultimately leading to widespread tissue damage [6]. Elevated levels of pro-inflammatory cytokines, particularly interleukin-1β (IL-1β), interleukin-6 (IL-6), tumor necrosis factor (TNF), and interferon gamma-induced protein 10 (IP-10), have been reported in individuals with long COVID [7,8]. Dysregulated immune responses—especially cytokine release syndrome—can result in severe cytotoxicity and multiorgan dysfunction, affecting the cardiovascular, respiratory, central nervous, renal, and hepatic systems, thereby increasing morbidity and mortality [9,10]. Clinically, individuals with long COVID commonly report a broad spectrum of persistent symptoms, including fatigue, exertional dyspnea, and reduced exercise capacity [11].

Impaired exercise performance is one of the more modifiable complications observed in individuals with long COVID [11]. Emerging evidence indicates that individualized physical exercise programs can significantly support functional recovery in many patients [12,13]. For those without post-exertional malaise, recommended interventions include continuous endurance training, interval training, resistance training, and respiratory muscle strength training, typically performed 3–5 times per week. Among these, high-intensity interval training (HIIT)—which involves short bursts of intense activity—has been shown to be both safe and as effective as continuous aerobic exercise. HIIT is commonly prescribed at 80–100% of maximal intensity or a perceived exertion level of 5–6 on a 10-point scale [12]. This training modality has been associated with improvements in mental well-being, increased cardiac muscle mass, enhanced renal function, and improved exercise tolerance [14,15,16]. However, in individuals with chronic fatigue syndrome or post-exertional malaise, exercise may exacerbate symptoms, and the current evidence suggests that physical activity can worsen the condition in a substantial proportion of long COVID patients [12]. Therefore, further research is warranted to elucidate the underlying mechanisms and determine safe and effective HIIT protocols for this population.

Nutritional supplementation may complement structured exercise by supporting antioxidant defenses and immune function [17,18,19]. Herbal preparations, including *Andrographis paniculata* and white fingerroot (*Boesenbergia rotunda*), have received growing attention for their potential roles in the prevention and treatment of COVID-19 [20,21]. *Triphala*, a traditional Ayurvedic formulation comprising *Emblica officinalis* (Indian gooseberry), *Terminalia chebula*, and *Terminalia bellirica*, has been reported in preclinical models to exert antioxidant and anti-inflammatory effects [22,23,24]. While these findings suggest potential supportive roles, evidence in the context of long COVID remains limited.

Our review of the literature revealed a lack of studies examining the effects of *Triphala* supplementation in individuals with long COVID. Most of the existing research has focused on its application during the acute phase of SARS-CoV-2 infection. Although HIIT is a well-established intervention for improving physical fitness in patients without post-exertional malaise, the mechanisms by which HIIT modulates inflammation and oxidative stress in individuals with long COVID—particularly those experiencing post-exertional malaise—remain poorly understood. Furthermore, to date, no studies have explored the combined effects of HIIT and *Triphala* supplementation in this population. Therefore, this study primarily aimed to examine the effects of an 8-week HIIT program on inflammation, oxidative stress, and exercise-related symptoms in individuals with long COVID. A secondary objective was to explore whether adjunct *Triphala* supplementation confers additional benefits. The findings may provide evidence-based guidance for integrating HIIT into comprehensive rehabilitation strategies for individuals with long COVID, while also offering preliminary insights into the potential adjunctive role of *Triphala*.

## 2. Results

### 2.1. Participant Characteristics

Of the 112 individuals initially enrolled in the study, 8 participants did not complete the intervention: 5 contracted COVID-19 during the study period, 1 experienced unrelated health issues, and 2 were unable to attend the post-intervention assessment (one from the control group and one from the combined group). As a result, data from 104 participants were included in the final analysis. The sample comprised 28 males (23%) and 76 females (77%), with a mean age of 22.41 ± 6.85 years. At baseline, there were no statistically significant differences among the three groups in terms of age, sex, body mass (BM), body mass index (BMI), frequency of COVID-19 infection, or recovery time from the most recent infection (*p* > 0.05), indicating good comparability in physical and clinical characteristics. The only exception was height, which was significantly greater in the combined group compared to the HIIT group (*p* = 0.015) (Table 1).

Participants in the HIIT group exercised at a maximal cycling load of 2.08 ± 0.39 kp and a minimal load of 0.92 ± 0.26 kp. In the combined group, the maximal and minimal cycling loads were 2.31 ± 0.65 kp and 0.99 ± 0.24 kp, respectively. There were no statistically significant differences in cycling loads between the two groups. Breathlessness decreased in both groups during recovery (HIIT: −2.61, 95% CI 1.99–3.24; combined: −2.13, 95% CI 1.60–2.67), with no significant between-group differences. Leg fatigue also remained unchanged and comparable between groups.

The exercise compliance rates were 71.32% ± 25.05% for the HIIT group and 78.27% ± 31.23% for the combined group. No serious adverse events were reported during the intervention period. Minor adverse events—including leg muscle fatigue, dizziness, and transient hypotension—were observed in four participants (5.97%) during the final week of the intervention, specifically during the fourth HIIT cycle. Despite these occurrences, no participants discontinued the exercise program.

### 2.2. Inflammatory Biomarkers

**Figure 1:**
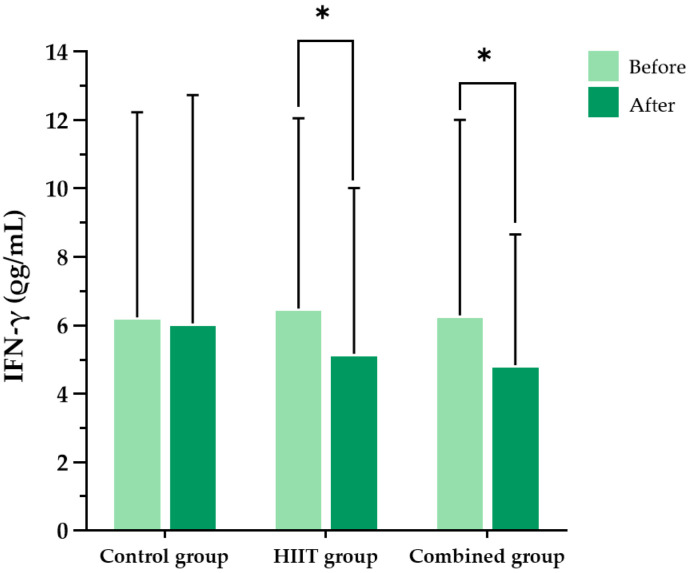
Blood interferon-gamma (IFN-γ) levels in the three participant groups before and after the 8-week intervention. Data are presented as the mean ± standard deviation (SD); *n* = 104. HIIT, high-intensity interval training; * *p* < 0.05 compared with baseline (within-group comparison).

**Figure 2:**
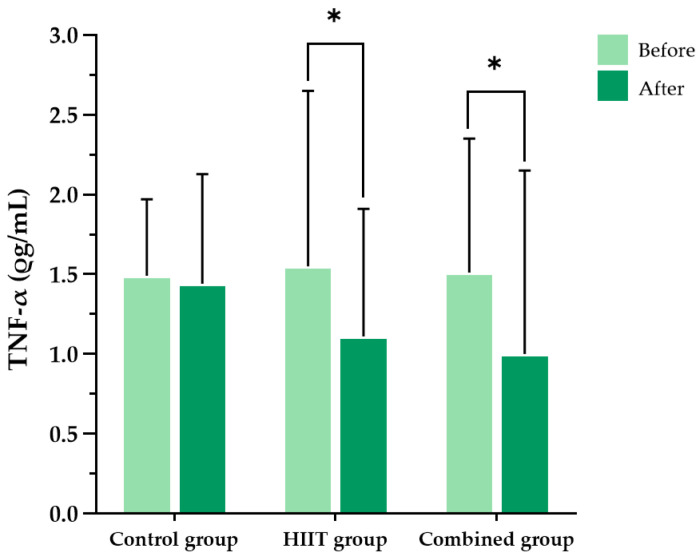
Blood tumor necrosis factor-alpha (TNF-α) levels in the three participant groups before and after the 8-week intervention. Data are presented as the mean ± standard deviation (SD); *n* = 104. HIIT, high-intensity interval training; * *p* < 0.05 compared with baseline (within-group comparison).

At baseline, there were no significant differences in interferon-gamma (IFN-γ) or tumor necrosis factor-alpha (TNF-α) concentrations among the three groups. Following the 8-week intervention, significant within-group reductions in both IFN-γ and TNF-α levels were observed in the HIIT group (*p* = 0.047 and *p* < 0.001, respectively) and the combined group (*p* = 0.033 and *p* < 0.001, respectively). In contrast, no significant changes were detected in the control group for IFN-γ (*p* = 0.860) or TNF-α (*p* = 0.496). However, between-group comparisons using repeated-measures one-way analysis of variance (ANOVA) revealed no statistically significant group-by-time interaction effects for either IFN-γ (F (2, 36) = 0.179, partial η^2^ = 0.010, *p* = 0.837) or TNF-α (F (2, 8) = 2.189, partial η^2^ = 0.354, *p* = 0.175), indicating that the observed reductions were not significantly different between groups (Figure 1 and Figure 2).

### 2.3. Oxidative Stress and Antioxidative Biomarkers

**Figure 3:**
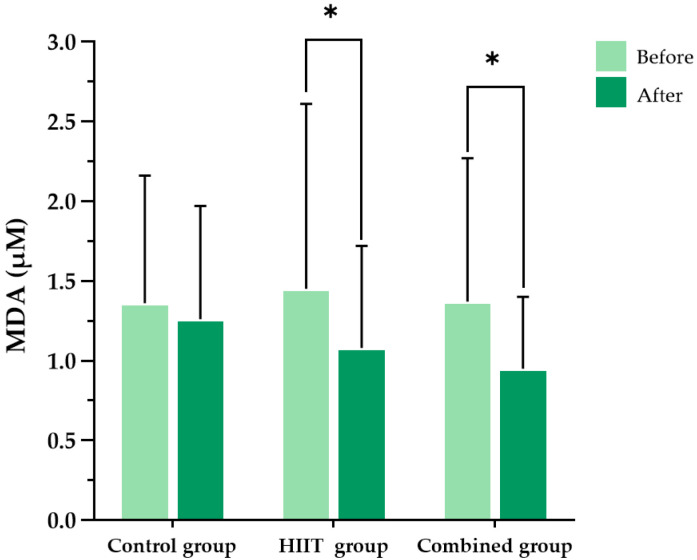
Blood malondialdehyde (MDA) levels in the three participant groups before and after the 8-week intervention. Data are presented as the mean ± standard deviation (SD); *n* = 104. HIIT, high-intensity interval training; * *p* < 0.05 compared with baseline (within-group comparison).

**Figure 4:**
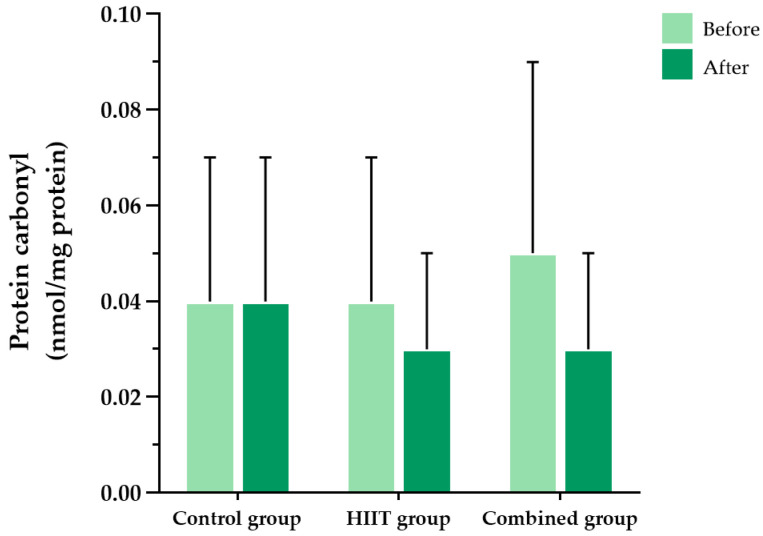
Blood protein carbonyl levels in the three participant groups before and after the 8-week intervention. Data are presented as the mean ± standard deviation (SD); *n* = 104. HIIT, high-intensity interval training.

At baseline, there were no significant differences among the three groups in malondialdehyde (MDA), protein carbonyls, or superoxide dismutase (SOD) activity levels. Following the 8-week intervention, significant within-group reductions in both MDA and protein carbonyl levels were observed in the HIIT group (*p* = 0.037 and *p* = 0.047, respectively) and the combined group (*p* = 0.014 and *p* = 0.018, respectively). No significant changes were observed in the control group for MDA (*p* = 0.636) or protein carbonyls (*p* = 0.899). However, between-group comparisons revealed no statistically significant group-by-time interaction effects for MDA (F (2, 56) = 0.345, partial η^2^ = 0.012, *p* = 0.710) or protein carbonyls (F (2, 38) = 0.032, partial η^2^ = 0.002, *p* = 0.968) (Figure 3 and Figure 4).

**Figure 5:**
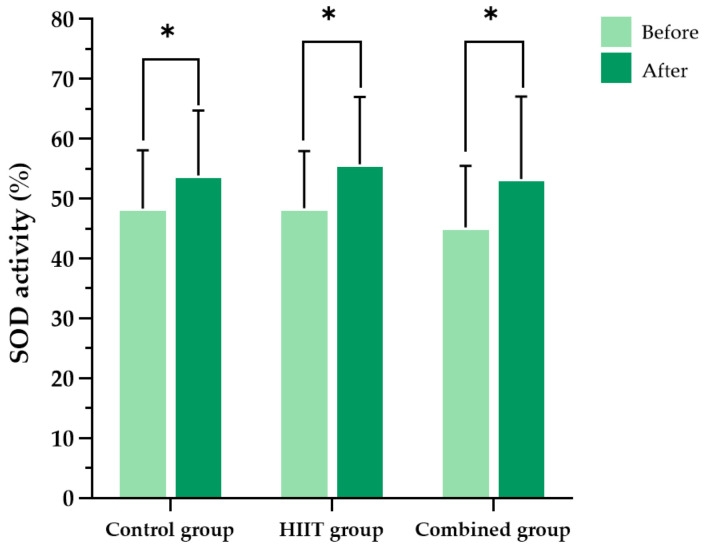
Blood superoxide dismutase (SOD) activity levels in the three participant groups before and after the 8-week intervention. Data are presented as the mean ± standard deviation (SD); *n* = 104. HIIT, high-intensity interval training; * *p* < 0.05 compared with baseline (within-group comparison).

Additionally, SOD activity significantly increased in all groups after the intervention (*p* < 0.001). Nevertheless, no significant differences were observed between groups (F (2, 62) = 0.610, partial η^2^ = 0.019, *p* = 0.546) (Figure 5).

### 2.4. Liver and Kidney Function Biomarkers

No significant within-group changes were observed in either alanine aminotransferase (ALT) (*p* = 0.315) or creatinine (*p* = 0.249), indicating that *Triphala* supplementation did not adversely affect liver or kidney function over the 8-week intervention period.

## 3. Discussion

This study demonstrated that an 8-week HIIT program significantly reduced pro-inflammatory cytokines (IFN-γ, TNF-α), oxidative stress markers (MDA, protein carbonyls), and perceived exertion in individuals with long COVID. These findings highlight the potential of HIIT as an effective and feasible rehabilitation strategy for addressing persistent inflammation, oxidative stress, and exercise intolerance, which are among the most burdensome sequelae of long COVID.

In addition to its well-established cardiovascular and metabolic benefits, HIIT has been reported to enhance antioxidant capacity, reduce oxidative damage to mitochondrial DNA, and stimulate mitochondrial biogenesis, thereby contributing to improved redox homeostasis [25]. Consistent with these findings, Delwing-de Lima et al. [26] reported that HIIT reduces oxidative stress markers such as thiobarbituric acid-reactive substances and protein carbonyls, while simultaneously increasing antioxidant enzymes, including catalase and SOD. These observations align with our observations, in which significant reductions in MDA and protein carbonyl levels were evident in both the HIIT-only and HIIT plus *Triphala* groups.

Emerging evidence also indicates that HIIT modulates immune responses and attenuates systemic inflammation. Malczyńska-Sims et al. [27] reported that a 12-week HIIT program significantly reduced TNF-α levels and increased anti-inflammatory cytokine IL-10 levels, alongside elevated SOD activity. These immunoregulatory and anti-inflammatory effects are consistent with our findings, in which both intervention groups demonstrated significant reductions in IFN-γ and TNF-α levels.

An 8-week intervention period was selected because previous studies have shown that cardiorespiratory fitness, oxidative stress markers, and metabolic regulation can improve within 6 to 8 weeks of structured HIIT [28,29,30]. In addition, Panahi et al. [31] reported that 8 weeks of curcuminoid supplementation reduced oxidative stress in patients with metabolic syndrome, further supporting the appropriateness of this timeframe for evaluating antioxidant outcomes. While a recent trial demonstrated the benefits of a 12-week HIIT intervention in preserving cardiac structure in post-COVID-19 patients [32], the choice of an 8-week duration in the present study balanced clinical efficacy with feasibility and adherence, particularly given the post-exertional fatigue and reduced tolerance common in long COVID.

Exercise-based rehabilitation programs lasting 4 to 8 weeks have been shown to reduce breathlessness and fatigue in long COVID patients, with aerobic and interval-based exercises producing favorable outcomes [33]. These findings are consistent with our results, in which dyspnea (measured by perceived exertion) improved significantly after 8 weeks of HIIT. Moreover, longer protocols such as a 12-week HIIT program have shown additional benefits for post-COVID-19 cardiac structure and function [32], further supporting both the safety and efficacy of HIIT in this population.

An 8-week exercise intervention in COVID-19 survivors has also been shown to reduce pro-inflammatory markers such as IL-6 and fibrinogen, alongside improvements in physical performance [34]. Consistent with these findings, our study found within-group reductions in IFN-γ and TNF-α in both of the intervention groups, suggesting that HIIT may attenuate chronic low-grade inflammation in individuals with long COVID.

Although direct evidence from 8-week HIIT studies on oxidative stress in COVID-19 is limited, data from other populations show that 3 to 8 weeks of HIIT reduces lipid peroxidation markers while increasing antioxidant enzyme activity. For example, Bogdanis et al. [35] reported that just 6 weeks of HIIT attenuated oxidative stress responses and improved antioxidant capacity in healthy individuals. These mechanisms likely explain the reductions in MDA and protein carbonyls observed in our HIIT and combined groups. A recent review also concluded that HIIT functions as a form of “redox medicine,” capable of restoring redox balance across cardiometabolic populations [36].

The mechanisms underlying the benefits of HIIT are multifactorial. First, HIIT promotes anti-inflammatory effects by modulating cytokine signaling. Repeated high-intensity exercise stimulates the production of anti-inflammatory mediators such as IL-10 and downregulates pro-inflammatory cytokines, including TNF-α and IL-6, thereby restoring immune homeostasis [37]. In post-COVID-19 patients, HIIT has been shown to significantly reduce circulating inflammatory markers, suggesting improved immune regulation [38]. Second, HIIT enhances antioxidant defense systems. Transient increases in reactive oxygen species during exercise activate redox-sensitive transcription factors such as nuclear factor erythroid 2-related factor 2, which upregulates endogenous antioxidants, including SOD and glutathione peroxidase [39]. This hormetric adaptation reduces oxidative damage over time. Clinical studies in post-COVID-19 populations have demonstrated that 8 weeks of structured HIIT improved antioxidant enzyme activity and reduced markers of lipid peroxidation, such as MDA [38]. Finally, HIIT improves vascular health and mitochondrial function, both of which are impaired in the context of long COVID. Enhanced mitochondrial biogenesis increases oxidative phosphorylation efficiency and decreases excess ROS production [40], while improved endothelial function through increased nitric oxide bioavailability further reduces oxidative stress and systemic inflammation [41]. Together, these adaptations contribute to reductions in fatigue, dyspnea, and exercise intolerance commonly reported in individuals with long COVID.

In this study, SOD levels increased across all experimental groups, even after controlling for potential confounders. The participants were instructed to maintain their usual physical activity and dietary habits throughout the study period, and to obtain at least seven hours of sleep per night, particularly on the night before the post-test. However, certain lifestyle factors that could influence antioxidant status—such as alcohol consumption, environmental exposures (e.g., pollutants, ultraviolet radiation), and medication use—were not restricted, in order to preserve ecological validity [42].

*Triphala* supplementation, while safe and well tolerated, did not provide additional statistically significant benefits beyond HIIT; its anti-inflammatory and antioxidant effects are likely attributable to its rich phytochemical profile. Prior studies have identified polyphenols and tannins, including gallic acid, tannic acid, syringic acid, and epicatechin, as key compounds contributing to *Triphala*’s biological activity [43,44]. *Triphala* also contains vitamin C (ascorbic acid), chebulagic acid, and chebulinic acid, which have shown antioxidant, anti-inflammatory, cytoprotective, and antimicrobial effects in preclinical models. While these compounds may exert additive or synergistic actions, the incremental effects were not statistically significant in our trial. Nonetheless, the favorable safety profile suggests that *Triphala* remains a candidate for further study as an adjunct nutritional therapy, particularly in populations that are unable to fully engage in high-intensity exercise.

The broad range of active constituents in our *Triphala* preparation may partially explain the trends toward greater reductions in inflammatory and oxidative stress markers observed in the combined group, despite the absence of between-group statistical significance. These findings support the notion that *Triphala*’s efficacy is derived from the interplay of multiple phytochemicals acting through complementary mechanisms. The phytochemical composition identified in this study aligns with prior reports and highlights additional compounds with antioxidant, anti-inflammatory, immunomodulatory, and cytoprotective properties. Collectively, these observations reinforce *Triphala*’s therapeutic potential as a complementary intervention for modulating inflammation and oxidative stress in long COVID.

Importantly, no evidence of hepatic or renal toxicity was observed following *Triphala* supplementation. Serum ALT and creatinine remained stable in the combined group, indicating that administration at 1000 mg/day, five days per week for eight weeks, was safe and well tolerated. These findings support *Triphala*’s continued investigation as a low-risk adjunct to structured exercise rehabilitation programs.

This study has several limitations that warrant consideration. First, the sample size was calculated based on expected changes in MDA observed in a different population. While this provided a practical framework for trial design, it may not have been sufficiently sensitive to detect clinically relevant changes in long COVID, potentially leading to type II error. Future trials should consider larger sample sizes based specifically on biomarker responses in long COVID populations. Second, although this study comprehensively assessed inflammatory and oxidative stress biomarkers, it did not include patient-reported outcomes related to post-exertional symptom exacerbation (PESE) within 24 h of exercise, which is recommended in the evaluation of long COVID. Such measures would have provided important insight into the tolerability of HIIT interventions in this patient group. Third, while the participants were instructed to maintain stable dietary and lifestyle habits during the study, uncontrolled variables such as alcohol consumption, environmental exposures, and concurrent medication use may have influenced oxidative stress and inflammatory outcomes. These factors represent unavoidable sources of confounding in free-living populations. Fourth, although a panel of biomarkers was assessed, the scope was limited. Key markers such as IL-6, IL-10, and glutathione peroxidase, as well as direct measures of mitochondrial function, were not included. Expanding biomarker profiling in future studies would allow for a more comprehensive evaluation of the mechanistic pathways through which HIIT and *Triphala* exert their effects. Finally, the *Triphala* dose selected (1000 mg/day, five days per week) was based on prior evidence suggesting a safe human dosage range of 1000–2000 mg/day. As this was an early-stage trial, the lowest dose was chosen to ensure safety and tolerability. However, this conservative dosing strategy may have limited the magnitude of the therapeutic effects observed. Subsequent studies should explore higher dosages in a *Triphala*-only group within the recommended range, in order to determine whether greater efficacy can be achieved without compromising safety.

## 4. Materials and Methods

### 4.1. Study Design: Randomized and Non-Blinded

**Figure 6:**
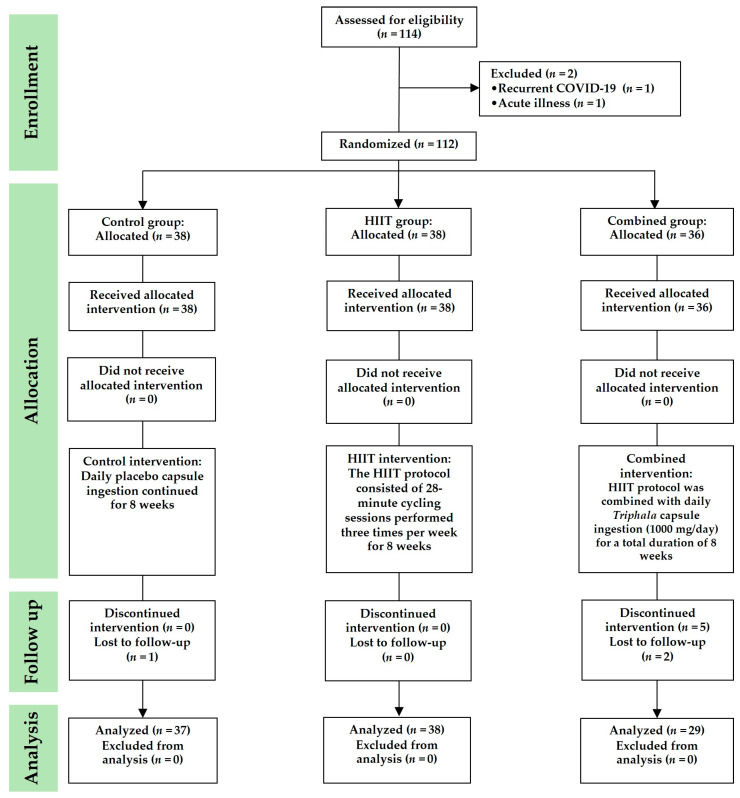
CONSORT flow diagram.

This randomized controlled trial was conducted between June and October 2023. The participants were assigned to study groups using a simple randomization method involving a sealed, closed-envelope draw. Each envelope was labeled to represent one of the three study groups, with the total number of envelopes corresponding to the number of participants. Randomization was conducted in the presence of a research assistant, who also confirmed each participant’s willingness and availability to participate. The participants were randomly allocated to one of three groups: (1) control group, (2) HIIT group, and (3) combined group (HIIT + *Triphala* supplementation) (Figure 6). This study was conducted using a double-blind design. Participants assigned to the HIIT, combined, or control group were blinded to their group allocation and were informed only that they would be participating in a bicycle exercise study. This approach minimized expectation bias and helped to ensure that the participants’ behavior was not influenced by knowledge of their group assignment. Three researchers (T.P., S.I. and P.P.) were responsible for participant screening, data collection, and data analysis. Biochemical analyses were performed by two researchers (O.B. and Y.T.), while immunological assays were conducted by certified medical technicians (N.P. and S.B.). All laboratory investigators (O.B., Y.T., N.P. and S.B.) who analyzed biological samples were blinded to the group allocation. Run identification codes were generated sequentially according to the order of participant enrollment, and these codes contained no information that could indicate whether a sample originated from the HIIT, combined, or control group. This procedure ensured that the investigators remained unaware of the group assignments throughout the study, thereby reducing the risk of measurement or analytical bias.

This study was approved by the Institutional Review Board of Burapha University (Approval No. IRB1-050/2566) and was registered at ClinicalTrials.gov (Registration No. NCT06208761). All of the participants were fully informed about the study procedures and provided written informed consent prior to enrollment, in accordance with the Declaration of Helsinki.

### 4.2. Screening of Participants

Eligible participants were male or female adults aged 18–59 years with a BMI between 18.5 and 24.9 kg/m^2^. All of the participants had previously tested positive for COVID-19 or had recovered from COVID-19 for at least four weeks and were free of active infection at the time of enrollment. Additionally, the participants exhibited persistent symptoms commonly associated with long COVID and post-exertional malaise, including fatigue, headache, difficulty concentrating, hair loss, and shortness of breath. The exclusion criteria included the following: (1) engaging in regular physical activity, defined as ≥2 sessions per week or ≥150 min per week; (2) regular use of dietary supplements; (3) smoking; (4) regular alcohol consumption, defined as more than one standard drink per day; (5) known food allergies, particularly to herbal ingredients such as *Emblica officinalis*, *Terminalia chebula*, or *Terminalia bellirica*; and (6) presence of any underlying medical conditions, including hypertension, diabetes, cardiovascular disease, cerebrovascular disease, respiratory disease, musculoskeletal disorders, cancer, liver or kidney disease, thyroid dysfunction, immune-related disorders, or other infectious diseases. Participants were also excluded if they had abnormal white blood cell counts or showed signs of active infection or inflammation, such as fever (>37.5 °C), pain, swelling, or redness. Termination criteria included the following: (1) initiation of dietary supplement use during the study period, (2) failure to comply with at least 80% of the prescribed exercise sessions, or (3) withdrawal of consent at any point during the study.

### 4.3. Sample Size

The sample size for this study was calculated using a formula for comparing means across more than two groups [45]. Due to the limited clinical data on the effects of *Triphala* and HIIT on oxidative stress in post-COVID-19 patients, the calculation was based on a previous study in patients with metabolic syndrome who received curcuminoid extract, a polyphenolic compound, at a dosage of 1 g/day for 8 weeks. That study reported a significant reduction in MDA levels (15.62 ± 2.59 nmol/mL) compared with the placebo group (20.16 ± 3.11 nmol/mL) [31]. Using these values, the required sample size was estimated to be 29.04 participants per group. To account for a potential dropout rate of 20%, the sample size was increased to at least 36 participants per group, resulting in a total of 108 participants across the three study groups.

### 4.4. High-Intensity Interval Training (HIIT)

In this study, we selected a cycling-based HIIT protocol consisting of four 3-min intervals at 85% of peak heart rate (HRpeak), interspersed with 3-min active recovery periods at 60% HRpeak. This format was chosen because it has been shown to be safe, feasible, and effective in improving cardiorespiratory fitness and metabolic regulation in both cardiometabolic and post-viral populations. Compared with other HIIT modalities, such as Tabata-style training or combined aerobic–resistance protocols, the 4 × 3 format provides a more standardized and tolerable approach, reducing the risk of excessive fatigue while still eliciting robust physiological adaptations [28,30]. This rationale supported our decision to adopt the 4 × 3 min HIIT cycling protocol in individuals with long COVID.

HIIT was defined as stationary cycling on a Monark 828E Ergomedic cycle ergometer (Monark, Sweden) at 85% of HRpeak for 4 min, alternating with light-intensity cycling at 60% of HRpeak for 3 min, following a 4:3 work-to-recovery ratio [12]. This cycle was repeated four times, resulting in a total session duration of 28 min. The participants performed HIIT three times per week over an 8-week period [29,30]. All exercise sessions were conducted in the MS 204 Physiotherapy Research Laboratory. Each session was supervised by two licensed physical therapists (T.P. and P.P.) to ensure participant safety, monitor exertion levels, and ensure adherence to proper exercise technique.

On the first day of the study, participants in the HIIT and combined groups underwent HRpeak assessment during cycling using the YMCA cycle ergometry protocol [46] to determine individualized target training intensities. Exercise sessions were scheduled according to each participant’s availability. Each session began with a 5-min unloaded warm-up on a stationary bicycle. Resistance was then adjusted to achieve 85% of the participant’s HRpeak during the 4-min high-intensity intervals, with a target cadence of 50–60 revolutions per minute (rpm). Each high-intensity bout was followed by a 3-min active recovery phase at 60% of HRpeak. This 4:3 work-to-recovery cycle was repeated four times, followed by a 5-min cool-down period. The participants were continuously monitored throughout each session for any signs of adverse events. Post-exercise assessments were conducted by the supervising research team to ensure safety and protocol adherence.

Post-exertional dyspnea was evaluated using the Borg rating of perceived exertion scale [47]. This numerical scale ranges from 6 (no exertion) to 20 (maximum effort). Scores of 7–11 correspond to extremely light to light exertion, 12–14 indicates moderate to somewhat hard exertion, and 15–17 represents hard to very hard exertion. The degree of leg fatigue was assessed after 10 min of cycling using a visual analog scale ranging from 0 (no fatigue) to 10 (maximal fatigue) [48]. On the first day of data collection, the participants were provided with a detailed explanation of the scale to ensure accurate self-reporting. During the exercise sessions, researchers (T.P. and P.P.) recorded individual RPE scores to monitor post-exercise exertion.

### 4.5. Triphala Supplement

In this study, *Triphala* was administered in capsule form. The capsules were manufactured by Charoen Suk Pharma Supply Co., Ltd., a GMP-certified pharmaceutical facility located in Mueang Nakhon Pathom, Nakhon Pathom Province, Thailand. The preparation process involved washing ripe fruits of *Emblica officinalis*, *Terminalia chebula*, and *Terminalia bellirica*, which were then thinly sliced and dried in a hot-air oven at 45–50 °C for 48 h. The dried materials were confirmed to have a moisture content of less than 10%. Subsequently, the dried fruits were ground into a fine powder and passed through a No. 80–100 mesh sieve to ensure uniform particle size. The powder was then encapsulated using an automatic capsule-filling machine. Each size-0 capsule contained 500 mg of the standardized *Triphala* formulation.

Each *Triphala* capsule contained *Emblica officinalis*, *Terminalia chebula*, and *Terminalia bellirica* at a ratio of 12:4:8, as recommended in the Thai Medicinal Properties Handbook for Winter Medicine. This traditional formulation is believed to relieve excess phlegm (Apothathu) and is considered to be appropriate for the prevention, treatment, and recovery phases of COVID-19. Quality control procedures were conducted in accordance with United States Pharmacopeia (USP) 36 guidelines for capsule weight variation. An initial sample of 20 capsules was assessed, with acceptable weight defined as 90–110% of the average capsule weight. If more than 2 capsules fell outside this range, an additional 40 capsules were tested. The batch was accepted if no more than 2–6 capsules deviated by more than 10% and none deviated by more than 25%. The capsules were packaged in opaque, properly labeled containers to ensure product stability, minimize photodegradation, and maintain traceability.

The chemical constituents of *Triphala* powder were identified using gas chromatography–mass spectrometry. The analysis was performed with an Agilent 7890A gas chromatograph coupled with an Agilent 7000B mass spectrometer (Agilent Technologies, Santa Clara, CA, USA) equipped with an HP-5 capillary column (30 m × 0.32 mm i.d., 0.25 µm film thickness). Briefly, 100 mg of *Triphala* powder was dissolved in 1 mL of ethanol. A 2 µL aliquot of the sample was injected using an autosampler in split mode (5:1), with an injection temperature of 250 °C and helium as the carrier gas at a flow rate of 1.0 mL/min. The oven temperature program was initiated at 40 °C (held for 5 min), then ramped to 200 °C over 25 min, and further increased to 280 °C over 61 min. The mass spectrometer was operated in electron ionization mode with an ion source temperature of 230 °C, electron energy of 70 eV, and a scan range of 50–650 *m*/*z*. Compound identification was conducted using GC-QQQ software (2020) in conjunction with the NIST MS Search 2.0 library (National Institute of Standards and Technology, Gaithersburg, MD, USA).

The major constituents identified in *Triphala* powder were 1,2,3-benzenetriol (48.82%), 5-hydroxymethylfurfural (2.21%), and catechol (2.06%). Additional phytochemical constituents and their respective relative abundances (%) included methyl salicylate (0.10%), butorphanol (0.13%), γ-tocopherol (0.12%), dl-α-tocopherol (0.37%), stigmasterol (0.26%), β-sitosterol (3.71%), lupeol (0.32%), oleic acid (8.33%), linoleic acid (6.52%), and squalene (0.05%). Many of these compounds are recognized for their pharmacological properties, including anti-inflammatory, antioxidant, and lipid-modulating activities. Their presence provides a mechanistic basis for the potential therapeutic effects of *Triphala*, particularly in mitigating persistent inflammation and oxidative stress during post-COVID-19 rehabilitation.

Based on the literature review, the appropriate dose of *Triphala* for humans with long COVID is estimated to be 1000–2000 mg/day in powdered capsule form. This dosage is considered to be suitable for targeting the mechanisms implicated in long COVID, including antioxidant, anti-inflammatory, metabolic-regulatory, and cardioprotective effects. However, the total daily intake should not exceed 4000 mg/day, as there are currently no safety data supporting higher doses in humans. Therefore, this study selected a dose of 1000 mg/day, with safety considerations as the primary rationale [22,23,49].

### 4.6. Experimental Protocols

The participants in each group were instructed to adhere to the following protocols over the 8-week intervention period:

Control Group: Participants did not engage in any structured exercise program or receive active supplementation. They were provided with a placebo capsule identical in size, shape, and appearance to the *Triphala* supplement, to be taken once daily for 8 weeks.

HIIT Group: Participants in this group followed a supervised HIIT program consisting of alternating high- and low-intensity cycling intervals. Each session lasted 28 min and was conducted three times per week for 8 weeks.

Combined Group: Participants in this group received both the daily *Triphala* supplement (500 mg twice daily, totaling 1000 mg/day) and the same supervised HIIT cycling program as described for the HIIT group, performed over the 8-week period.

### 4.7. Study Endpoints

The primary outcomes of this study were inflammatory biomarkers, specifically IFN-γ and TNF-α. These were assessed at baseline and after the 8-week intervention period. Secondary outcomes included biomarkers of oxidative stress and antioxidant defense—namely, MDA, protein carbonyls, and SOD activity. These markers were also measured pre- and post-intervention. Additionally, indicators of renal and hepatic function, including ALT and serum creatinine, were analyzed to monitor the safety and potential adverse effects of the interventions.

**Figure 7:**
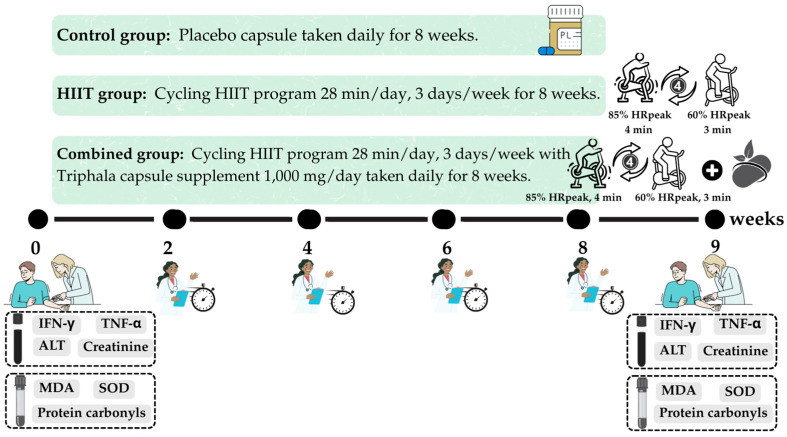
Experimental design and timeline of the 8-week intervention study. Blood samples were collected at baseline and after the intervention to assess inflammatory biomarkers (interferon-gamma (IFN-γ) and tumor necrosis factor-alpha (TNF-α)), oxidative stress markers (malondialdehyde (MDA) and protein carbonyls), and antioxidant defense (superoxide dismutase (SOD)). Liver and kidney functions were evaluated using serum alanine aminotransferase (ALT) and creatinine levels.

Blood samples were collected between 8:00 AM and 9:00 AM at the Exercise and Nutrition Innovation and Sciences Research Unit Laboratory. The timeline and sequence of assessments throughout the study period are presented in Figure 7.

#### 4.7.1. Interferon-Gamma (IFN-γ) and Tumor Necrosis Factor-Alpha (TNF-α)

Serum concentrations of IFN-γ and TNF-α were quantified using commercially available enzyme-linked immunosorbent assay (ELISA) kits, in accordance with the manufacturers’ protocols. Human IFN-γ (Cat. No. 3420-1HP-2, ELISA Pro: Human IFN-γ) and Human TNF-α (Cat. No. 3512-1HP-2, ELISA Pro: Human TNF-alpha) kits were obtained from Mebtech (Nacka, Sweden) and distributed by Biomed Diagnostics (Bangkok, Thailand). Optical density was measured at 450 nm using a microplate spectrophotometer (SpectraMax ABS, Molecular Devices LLC, San Jose, CA, USA). All assays were performed at the Medical Technology Laboratory. The standard curves demonstrated excellent linearity, with R^2^ values of 0.9991 for TNF-α and 0.9979 for IFN-γ, indicating high assay reliability.

#### 4.7.2. Malondialdehyde (MDA)

Plasma levels of MDA were measured in ethylenediaminetetraacetic acid (EDTA)-treated samples using HPLC with fluorescence detection, following a previously established protocol with some modifications [50]. This method involves the derivatization of MDA with TBA to form a fluorescent MDA-TBA adduct, improving specificity by minimizing interference from non-MDA TBA-reactive substances. Briefly, 150 μL of plasma was mixed with 10% trichloroacetic acid, 5 mM EDTA, 8% sodium dodecyl sulfate, and 500 ppm butylated hydroxytoluene. The mixture was incubated at room temperature for 10 min, followed by the addition of 535 μL of 0.6% TBA solution. The samples were then heated in a boiling water bath for 30 min and allowed to cool to room temperature. After cooling, the samples were centrifuged at 10,000 rpm for 5 min. The absorbance of the resulting supernatant was measured at 532 nm using a spectrophotometer. MDA concentrations were quantified against a standard curve generated using 1,1,3,3-tetraethoxypropane in the range of 0.33 to 9.89 μM. The assay demonstrated high linearity, with an R^2^ value of 0.98805.

#### 4.7.3. Protein Carbonyls

Protein carbonyl content, a marker of protein oxidation, was measured using a colorimetric assay based on the derivatization of carbonyl groups with 2,4-dinitrophenylhydrazine (DNPH), following the method of Levine et al. [51] with some modifications. Briefly, 400 μL of diluted plasma was divided into two microtubes: a sample tube and a control tube. The sample tube received 500 μL of 15 mM DNPH in 3.6 M hydrochloric acid (HCl), while the control received 500 μL of 3.6 M HCl alone. Both tubes were incubated in the dark at room temperature for 1 h, with vortexing every 15 min. Following incubation, 600 μL of 25% trichloroacetic acid (TCA) was added to each tube, and the samples were incubated on ice for 15 min before centrifugation at 10,000 rpm for 5 min at 4 °C. The supernatant was discarded, and the resulting protein pellet was washed once with 10% TCA and twice with a 1:1 mixture of ethyl acetate and ethanol. The pellet was then dried at 60 °C for 30 min and subsequently resuspended in 400 μL of 6 M guanidine hydrochloride. Samples were incubated at 37 °C with periodic vortexing and centrifuged at 2500 rpm for 10 min. The absorbance of 200 μL of the supernatant was measured at 360 nm using a microplate spectrophotometer to quantify protein carbonyls. Protein concentration was determined using the Bradford assay, with bovine serum albumin as the standard (0.05–1 mg/mL). Carbonyl content was expressed in nanomoles per milligram of protein (nmol/mg protein), using the following formula: protein carbonyl (nmol/mg protein) = (optical density at 360 nm × 80.16)/protein concentration (mg/mL).

#### 4.7.4. Superoxide Dismutase (SOD)

SOD activity, an indicator of antioxidant defense, was measured using a colorimetric assay with the SOD Assay Kit-WST (Dojindo Laboratories, Kumamoto, Japan), following the manufacturer’s protocol. Approximately 1 mL of clotted blood was collected from each participant, and serum was separated for analysis. This assay is based on the reduction of a water-soluble tetrazolium salt (WST-1) by superoxide anions, resulting in the formation of a water-soluble formazan dye. SOD inhibits this reaction by catalyzing the dismutation of superoxide radicals, and the degree of inhibition is directly proportional to the SOD activity in the sample. Absorbance was measured at the specified wavelength using a microplate spectrophotometer, and SOD activity was expressed in units per milliliter (U/mL).

#### 4.7.5. Alanine Aminotransferase (ALT) and Creatinine

Hepatic and renal functions were evaluated to monitor potential adverse effects of the 8-week *Triphala* supplementation. Blood samples collected in clot-activated tubes were analyzed for serum ALT and creatinine concentrations. Analyses were performed using the VITROS ALTV and VITROS CREA slide methods, respectively, following standard laboratory procedures at RIA Laboratory Co., Ltd., Chonburi, Thailand.

### 4.8. Statistical Analysis

Data distribution and homogeneity of variance were assessed using the Kolmogorov–Smirnov test. Between-group comparisons at baseline and post-intervention were conducted using one-way ANOVA, followed by Bonferroni post hoc tests. Within-group changes over time were analyzed using paired *t*-tests. To examine the interaction effects (group × time) and estimate effect sizes, a two-way repeated-measures ANOVA was conducted. Mauchly’s test of sphericity was applied, and partial eta-squared (η^2^) was reported as the measure of effect size. The Wilcoxon signed-rank test and the Mann–Whitney U test were applied to assess within-group changes and between-group differences in ordinal variables (specifically, breathlessness and leg fatigue levels). All statistical analyses were performed using IBM SPSS Statistics for Windows, version 26.0 (IBM Corp., Armonk, NY, USA). Data are presented as the mean ± standard deviation (mean ± SD), and a *p*-value of <0.05 was considered to be statistically significant.

## 5. Conclusions

This randomized controlled trial demonstrated that an 8-week HIIT program was effective in reducing inflammation, attenuating oxidative stress, and alleviating dyspnea in individuals with long COVID. These benefits were observed consistently in both the HIIT and HIIT + *Triphala* groups, with no statistically significant differences between them. *Triphala* supplementation was safe and well tolerated but did not provide additional measurable effects beyond those of HIIT. Taken together, these findings highlight HIIT as a feasible and evidence-based rehabilitation strategy for individuals with long COVID, while suggesting that the role of *Triphala* as an adjunct therapy requires further investigation.

## Figures and Tables

**Table 1:** Baseline physical and clinical characteristics of participants by group.

Characteristic	Control (*n* = 37)	HIIT (*n* = 38)	Combined (*n* = 29)
Age (years)	22.21 ± 6.81	20.76 ± 3.36	23.23 ± 7.86
Sex (male/female) (%)	9/28 (24/76)	7/31 (18/82)	12/17 (41/59)
Height (m)	1.64 ± 0.07	1.61 ± 0.07	1.66 ± 0.07 ^#^
Body mass (kg)	58.55 ± 11.99	54.88 ± 10.07	60.26 ± 10.36
Body mass index (kg/m^2^)	21.67 ± 3.37	21.09 ± 3.38	21.77 ± 2.86
COVID-19 infections (*n*)	1.21 ± 0.42	1.29 ± 0.52	1.47 ± 0.73
Recovery time (months)	8.52 ± 5.80	11.58 ± 5.75	9.45 ± 8.05
Rating of perceived exertion (Borg 6–20) (mode (range))			
Before		18 (13–20)	20 (15–20)
After		15 (13–18) *	18 (13–15) *
Visual analog scale of leg fatigue (VAS 0–10) (mode (range))			
Before		3 (2–5)	3 (2–7)
After		3 (2–8)	3 (2–10)

Data are presented as the mean ± SD or *n* (%). HIIT, high-intensity interval training. One-way analysis of variance (ANOVA) was used for continuous variables; chi-squared test was used for categorical variables; ^#^ *p* < 0.05 compared with the HIIT group. Wilcoxon signed-rank test was used for ordinal variables; * *p* < 0.05 compared with before in each group.

## Data Availability

The data are available upon request from the corresponding author.

## Abbreviations

The following abbreviations are used in this manuscript:

ALT	Alanine aminotransferase
BM	Body mass
BMI	Body mass index
COVID-19	Coronavirus disease 2019
DNA	Deoxyribonucleic acid
DNPH	Dinitrophenylhydrazine
EDTA	Ethylenediaminetetraacetic acid
HCl	Hydrochloric acid
HIIT	High-intensity interval training
HPLC	High-performance liquid chromatography
HR	Heart rate
IFN	Interferon
IL	Interleukin
IP-10	Interferon-gamma-induced protein 10
MDA	Malondialdehyde
SD	Standard deviation
SOD	Superoxide dismutase
TBA	Thiobarbituric acid
TCA	Trichloroacetic acid
TNF	Tumor necrosis factor

## References

[1] World Health Organization WHO COVID-19 Dashboard. https://data.who.int/dashboards/covid19/cases?n=c.

[2] Soriano J.B., Murthy S., Marshall J.C., Relan P., Diaz J.V. (2022). A clinical case definition of post-COVID-19 condition by a Delphi consensus. Lancet Infect. Dis..

[3] Ely E.W., Brown L.M., Fineberg H.V. (2024). Long Covid Defined. N. Engl. J. Med..

[4] Hastie C.E., Lowe D.J., McAuley A., Mills N.L., Winter A.J., Black C., Scott J.T., O’Donnell C.A., Blane D.N., Browne S. (2023). True prevalence of long-COVID in a nationwide, population cohort study. Nat. Commun..

[5] Davis H.E., McCorkell L., Vogel J.M., Topol E.J. (2023). Long COVID: Major findings, mechanisms and recommendations. Nat. Rev. Microbiol..

[6] Ortelli P., Ferrazzoli D., Sebastianelli L., Engl M., Romanello R., Nardone R., Bonini I., Koch G., Saltuari L., Quartarone A. (2021). Neuropsychological and neurophysiological correlates of fatigue in post-acute patients with neurological manifestations of COVID-19: Insights into a challenging symptom. J. Neurol. Sci..

[7] Peluso M.J., Lu S., Tang A.F., Durstenfeld M.S., Ho H.E., Goldberg S.A., Forman C.A., Munter S.E., Hoh R., Tai V. (2021). Markers of Immune Activation and Inflammation in Individuals With Postacute Sequelae of Severe Acute Respiratory Syndrome Coronavirus 2 Infection. J. Infect. Dis..

[8] Schultheiß C., Willscher E., Paschold L., Gottschick C., Klee B., Henkes S.S., Bosurgi L., Dutzmann J., Sedding D., Frese T. (2022). The IL-1β, IL-6, and TNF cytokine triad is associated with post-acute sequelae of COVID-19. Cell Rep. Med..

[9] Afrin L.B., Weinstock L.B., Molderings G.J. (2020). COVID-19 hyperinflammation and post-COVID-19 illness may be rooted in mast cell activation syndrome. Int. J. Infect. Dis..

[10] Tziolos N.-R., Ioannou P., Baliou S., Kofteridis D.P. (2023). Long COVID-19 Pathophysiology: What Do We Know So Far?. Microorganisms.

[11] Natarajan A., Shetty A., Delanerolle G., Zeng Y., Zhang Y., Raymont V., Rathod S., Halabi S., Elliot K., Shi J.Q. (2023). A systematic review and meta-analysis of long COVID symptoms. Syst. Rev..

[12] Gloeckl R., Zwick R.H., Fürlinger U., Schneeberger T., Leitl D., Jarosch I., Behrends U., Scheibenbogen C., Koczulla A.R. (2024). Practical Recommendations for Exercise Training in Patients with Long COVID with or without Post-exertional Malaise: A Best Practice Proposal. Sports Med. Open.

[13] Jia G., Su C.H. (2024). Tailored Physical Activity Interventions for Long COVID: Current Approaches and Benefits—A Narrative Review. Healthcare.

[14] Rasmussen I.E., Løk M., Durrer C.G., Foged F., Schelde V.G., Budde J.B., Rasmussen R.S., Høvighoff E.F., Rasmussen V., Lyngbæk M. (2023). Impact of high-intensity interval training on cardiac structure and function after COVID-19: An investigator-blinded randomized controlled trial. J. Appl. Physiol..

[15] Borrega-Mouquinho Y., Sánchez-Gómez J., Fuentes-García J.P., Collado-Mateo D., Villafaina S. (2021). Effects of High-Intensity Interval Training and Moderate-Intensity Training on Stress, Depression, Anxiety, and Resilience in Healthy Adults During Coronavirus Disease 2019 Confinement: A Randomized Controlled Trial. Front. Psychol..

[16] Pierros T., Spyrou K. (2023). Effects of high-intensity interval training versus sprint interval training during the second wave of COVID-19 lockdown on soccer players. Apunt. Sports Med..

[17] Cámara M., Sánchez-Mata M.C., Fernández-Ruiz V., Cámara R.M., Cebadera E., Domínguez L. (2021). A Review of the Role of Micronutrients and Bioactive Compounds on Immune System Supporting to Fight against the COVID-19 Disease. Foods.

[18] Sanduzzi Zamparelli S., Sanduzzi Zamparelli A., Bocchino M. (2025). Immune-Boosting and Antiviral Effects of Antioxidants in COVID-19 Pneumonia: A Therapeutic Perspective. Life.

[19] Mrityunjaya M., Pavithra V., Neelam R., Janhavi P., Halami P.M., Ravindra P.V. (2020). Immune-Boosting, Antioxidant and Anti-inflammatory Food Supplements Targeting Pathogenesis of COVID-19. Front. Immunol..

[20] Kanokkangsadal P., Mingmalairak C., Mukkasombat N., Kuropakornpong P., Worawattananutai P., Khawcharoenporn T., Sakpakdeejaroen I., Davies N.M., Itharat A. (2023). Andrographis paniculata extract versus placebo in the treatment of COVID-19: A double-blinded randomized control trial. Res. Pharm. Sci..

[21] Kanjanasirirat P., Suksatu A., Manopwisedjaroen S., Munyoo B., Tuchinda P., Jearawuttanakul K., Seemakhan S., Charoensutthivarakul S., Wongtrakoongate P., Rangkasenee N. (2020). High-content screening of Thai medicinal plants reveals Boesenbergia rotunda extract and its component Panduratin A as anti-SARS-CoV-2 agents. Sci. Rep..

[22] Bairwa V.K., Kashyap A.K., Meena P., Jain B.P. (2025). Triphala’s characteristics and potential therapeutic uses in modern health. Int. J. Physiol. Pathophysiol. Pharmacol..

[23] Rudrapal M., Celik I., Khan J., Ansari M.A., Alomary M.N., Yadav R., Sharma T., Tallei T.E., Pasala P.K., Sahoo R.K. (2022). Identification of bioactive molecules from Triphala (Ayurvedic herbal formulation) as potential inhibitors of SARS-CoV-2 main protease (Mpro) through computational investigations. J. King Saud. Univ. Sci..

[24] Chittasupho C., Umsumarng S., Srisawad K., Arjsri P., Phongpradist R., Samee W., Tingya W., Ampasavate C., Dejkriengkraikul P. (2024). Inhibition of SARS-CoV-2-Induced NLRP3 Inflammasome-Mediated Lung Cell Inflammation by Triphala-Loaded Nanoparticle Targeting Spike Glycoprotein S1. Pharmaceutics.

[25] Al-Rawaf H.A., Gabr S.A., Iqbal A., Alghadir A.H. (2023). High-Intensity Interval Training Improves Glycemic Control, Cellular Apoptosis, and Oxidative Stress of Type 2 Diabetic Patients. Medicina.

[26] Delwing-de Lima D., Ulbricht A., Werlang-Coelho C., Delwing-Dal Magro D., Joaquim V.H.A., Salamaia E.M., de Quevedo S.R., Desordi L. (2018). Effects of two aerobic exercise training protocols on parameters of oxidative stress in the blood and liver of obese rats. J. Physiol. Sci..

[27] Malczynska-Sims P., Chalimoniuk M., Wronski Z., Marusiak J., Sulek A. (2022). High-intensity interval training modulates inflammatory response in Parkinson’s disease. Aging Clin. Exp. Res..

[28] Weston M., Taylor K.L., Batterham A.M., Hopkins W.G. (2014). Effects of Low-Volume High-Intensity Interval Training (HIT) on Fitness in Adults: A Meta-Analysis of Controlled and Non-Controlled Trials. Sports Med..

[29] Sun F., Williams C.A., Sun Q., Hu F., Zhang T. (2024). Effect of eight-week high-intensity interval training versus moderate-intensity continuous training programme on body composition, cardiometabolic risk factors in sedentary adolescents. Front. Physiol..

[30] Gomes V.A., Fontoura F., Saquetto M.B., Ramos T., Santos S., Coutinho de Araujo W.S., Rivas P., Martinez B.P., Barreto A.P., Coelho Lima M.C. (2023). Comparison of High-Intensity Interval Training to Moderate-Intensity Continuous Training for Functioning and Quality of Life in Survivors of COVID-19 (COVIDEX): Protocol for a Randomized Controlled Trial. Phys. Ther..

[31] Panahi Y., Hosseini M.S., Khalili N., Naimi E., Majeed M., Sahebkar A. (2015). Antioxidant and anti-inflammatory effects of curcuminoid-piperine combination in subjects with metabolic syndrome: A randomized controlled trial and an updated meta-analysis. Clin. Nutr..

[32] Rasmussen I.E., Løk M., Durrer C.G., Lytzen A.A., Foged F., Schelde V.G., Budde J.B., Rasmussen R.S., Høvighoff E.F., Rasmussen V. (2024). Impact of a 12-week high-intensity interval training intervention on cardiac structure and function after COVID-19 at 12-month follow-up. Exp. Physiol..

[33] Mazzonetto L.F., Cordeiro J.F.C., Correia I.M., Oliveira A.d.S., Moraes C., Brilhadori J., Gomide E.B.G., Kudlacek M., Machado D.R.L., Anjos J.R.C.d. (2025). Physical Training Protocols for Improving Dyspnea and Fatigue in Long COVID: A Systematic Review with Meta-Analysis. Healthcare.

[34] Binabaji S., Rahimi M., Rajabi H., Keshavarz M., Rahimi R., Ahmadi A., Gahreman D. (2024). Effects of physical training on coagulation parameters, interleukin-6, and angiotensin-converting enzyme-2 in COVID-19 survivors. Sci. Rep..

[35] Carrizzo A., Forte M., Damato A., Trimarco V., Salzano F., Bartolo M., Maciag A., Puca A.A., Vecchione C. (2013). Antioxidant effects of resveratrol in cardiovascular, cerebral and metabolic diseases. Food Chem. Toxicol..

[36] Reljic D. (2025). High-Intensity Interval Training as Redox Medicine: Targeting Oxidative Stress and Antioxidant Adaptations in Cardiometabolic Disease Cohorts. Antioxidants.

[37] Batacan R.B., Duncan M.J., Dalbo V.J., Tucker P.S., Fenning A.S. (2017). Effects of high-intensity interval training on cardiometabolic health: A systematic review and meta-analysis of intervention studies. Br. J. Sports Med..

[38] Jimeno-Almazán A., Pallarés J.G., Buendía-Romero Á., Martínez-Cava A., Franco-López F., Sánchez-Alcaraz Martínez B.J., Bernal-Morel E., Courel-Ibáñez J. (2021). Post-COVID-19 Syndrome and the Potential Benefits of Exercise. Int. J. Environ. Res. Public Health.

[39] Done A.J., Traustadóttir T. (2016). Nrf2 mediates redox adaptations to exercise. Redox Biol..

[40] Little J.P., Safdar A., Bishop D., Tarnopolsky M.A., Gibala M.J. (2011). An acute bout of high-intensity interval training increases the nuclear abundance of PGC-1α and activates mitochondrial biogenesis in human skeletal muscle. Am. J. Physiol. Regul. Integr. Comp. Physiol..

[41] Li L., Liu X., Shen F., Xu N., Li Y., Xu K., Li J., Liu Y. (2022). Effects of high-intensity interval training versus moderate-intensity continuous training on blood pressure in patients with hypertension: A meta-analysis. Medicine.

[42] Papas A.M. (1996). Determinants of antioxidant status in humans. Lipids.

[43] Tarasiuk A., Mosińska P., Fichna J. (2018). Triphala: Current applications and new perspectives on the treatment of functional gastrointestinal disorders. Chin. Med..

[44] Prasad S., Srivastava S.K. (2020). Oxidative Stress and Cancer: Chemopreventive and Therapeutic Role of Triphala. Antioxidants.

[45] Rosner B.A. (2006). Fundamentals of Biostatistics.

[46] YMCA of the USA (2000). YMCA Fitness Testing and Assessment Manual.

[47] Borg G. (1970). Perceived exertion as an indicator of somatic stress. J. Rehabil. Med..

[48] Leung A.W., Chan C.C., Lee A.H., Lam K.W. (2004). Visual analogue scale correlates of musculoskeletal fatigue. Percept. Mot. Ski..

[49] Phimarn W., Sungthong B., Itabe H. (2021). Effects of Triphala on Lipid and Glucose Profiles and Anthropometric Parameters: A Systematic Review. J. Evid. Based Integr. Med..

[50] Nielsen F., Mikkelsen B.B., Nielsen J.B., Andersen H.R., Grandjean P. (1997). Plasma malondialdehyde as biomarker for oxidative stress: Reference interval and effects of life-style factors. Clin. Chem..

[51] Levine R.L., Garland D., Oliver C.N., Amici A., Climent I., Lenz A.G., Ahn B.W., Shaltiel S., Stadtman E.R. (1990). Determination of carbonyl content in oxidatively modified proteins. Methods Enzymol..

